# Propensity Score-Based Approaches to Confounding by Indication in Individual Patient Data Meta-Analysis: Non-Standardized Treatment for Multidrug Resistant Tuberculosis

**DOI:** 10.1371/journal.pone.0151724

**Published:** 2016-03-29

**Authors:** Gregory J. Fox, Andrea Benedetti, Carole D. Mitnick, Madhukar Pai, Dick Menzies, The Collaborative Group for Meta-Analysis of Individual Patient Data in MDR-TB

**Affiliations:** 1 Respiratory Epidemiology and Clinical Research Unit, McGill University, 2155 Guy St, Montreal, Quebec, H3H 2R9, Canada; 2 Harvard Medical School, 641 Huntington Avenue Boston, MA, 02115, United States of America; 3 Department of Epidemiology, Biostatistics & Occupational Health, McGill University, 1020 Pine Avenue West, Montreal, H3A 1A2, Canada; Ohio State University College of Medicine, UNITED STATES

## Abstract

**Background:**

In the absence of randomized clinical trials, meta-analysis of individual patient data (IPD) from observational studies may provide the most accurate effect estimates for an intervention. However, confounding by indication remains an important concern that can be addressed by incorporating individual patient covariates in different ways. We compared different analytic approaches to account for confounding in IPD from patients treated for multi-drug resistant tuberculosis (MDR-TB).

**Methods:**

Two antibiotic classes were evaluated, fluoroquinolones—considered the cornerstone of effective MDR-TB treatment—and macrolides, which are known to be safe, yet are ineffective *in vitro*. The primary outcome was treatment success against treatment failure, relapse or death. Effect estimates were obtained using multivariable and propensity-score based approaches.

**Results:**

Fluoroquinolone antibiotics were used in 28 included studies, within which 6,612 patients received a fluoroquinolone and 723 patients did not. Macrolides were used in 15 included studies, within which 459 patients received this class of antibiotics and 3,670 did not. Both standard multivariable regression and propensity score-based methods resulted in similar effect estimates for early and late generation fluoroquinolones, while macrolide antibiotics use was associated with reduced treatment success.

**Conclusions:**

In this individual patient data meta-analysis, standard multivariable and propensity-score based methods of adjusting for individual patient covariates for observational studies yielded produced similar effect estimates. Even when adjustment is made for potential confounding, interpretation of adjusted estimates must still consider the potential for residual bias.

## Introduction

In the absence of randomized clinical trials, data from non-experimental observational studies may provide the only data to evaluate complex medical interventions. Data from these observational studies can be pooled to provide an estimate of effect that may be more precise than that obtained by a single study [1, 2].

However traditional aggregate data meta-analyses, especially of observational studies, have limited capacity to adequately account for factors which confound the association between treatment and outcome [3]. In contrast, individual patient data (IPD) meta-analysis incorporates individual patient characteristics in the analysis, permitting adjustment for the same set of covariates across multiple studies [4, 5].

Traditionally, multivariable regression is used to account for differences in measured covariates between subjects. However, this method may not fully adjust for confounding by indication occurring if the health status of patients affects treatment allocation. Alternative analytic approaches based upon propensity scores have been proposed that may provide more precise estimates of the treatment effect in observational studies in which confounding by indication may occur [6]. Defined as the predicted probability (propensity) of being given treatment, this method can incorporate the measured covariates of individuals in a variety of ways [6–8]. Propensity score-based analytic methods have been widely used in individual studies. However, this method has not yet been operationalized for IPD meta-analyses.

Recommended combination antibiotic therapy for multi-drug resistant tuberculosis (MDR-TB) includes at least five antibiotics, and is given for at least 18 months [9]. Current international treatment recommendations are based entirely on analysis of observational studies, as there have been no randomized phase 3 trials of MDR-TB treatment [10]. Consequently, the selection of treatment of MDR-TB treatment is particularly challenging, with little evidence to guide decision-making. Medical treatment for MDR-TB is highly individualized, with clinicians selecting drug regimens based upon the severity of disease, antibiotic resistance patterns of the causative bacteria and their own local clinical experience. As a result, the baseline patient characteristics are likely to strongly influence treatment allocation.

Here we apply a number of methods to adjust for confounding by indication using a large IPD dataset of patients treated with combination antibiotic therapy for MDR-TB. In order to better evaluate how effective our analytic methods were in controlling confounding we selected two antibiotic classes believed to have very different efficacy in TB treatment: fluoroquinolone (FQN) and macrolide antibiotics.

Randomized trials have shown that FQN antibiotics are effective in the treatment of drug susceptible TB, as a part of combination antibiotic therapy [11–13]. Given that its mechanism is the same regardless of the resistance to most other antibiotics, FQNs are also considered effective in treating MDR-TB—with just 17% of patients with MDR-TB worldwide having FQN resistance [14]. As a result, FQNs have become well-established as the cornerstone of MDR-TB treatment in national [15, 16] and international guidelines [17].

As a counterpoint, the macrolide class of antibiotics is inactive against M. *tuberculosis in vitro* [18] with inconsistent evidence supporting their clinical effect [17]. Consequently, macrolides are considered a “Group 5” antibiotic, recommended only for subjects with advanced drug resistance and few treatment options.

Drawing upon these two contrasting antibiotics of differing effectiveness, we aimed to explore confounding by indication in the context of IPD meta-analysis.

## Material and Methods

This project was approved by the Research Ethics Board of the Montreal Chest Institute, McGill University Health Centre, and when deemed necessary by local ethics boards of originally approved studies.

### Description of Individual Patient Data Set

**Fig 1:**
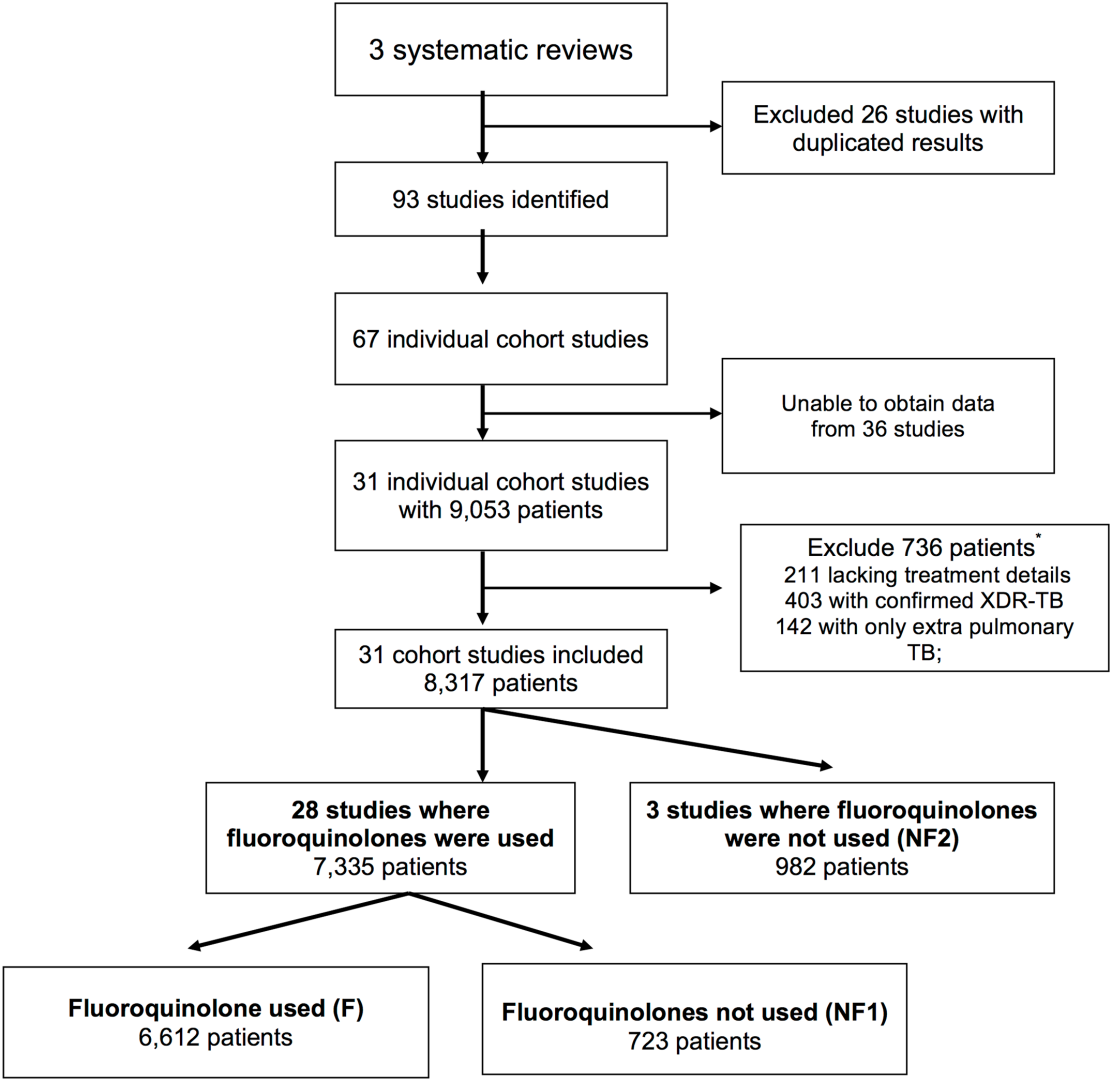
Consort diagram showing patient selection for an individual patient data meta-analysis of the effectiveness of fluoroquinolone antibiotics to treat multi-drug resistant tuberculosis. Group allocation was independent of which other antibiotics were concurrently used. *Some patients were excluded on the basis of more than one criterion.

**Fig 2:**
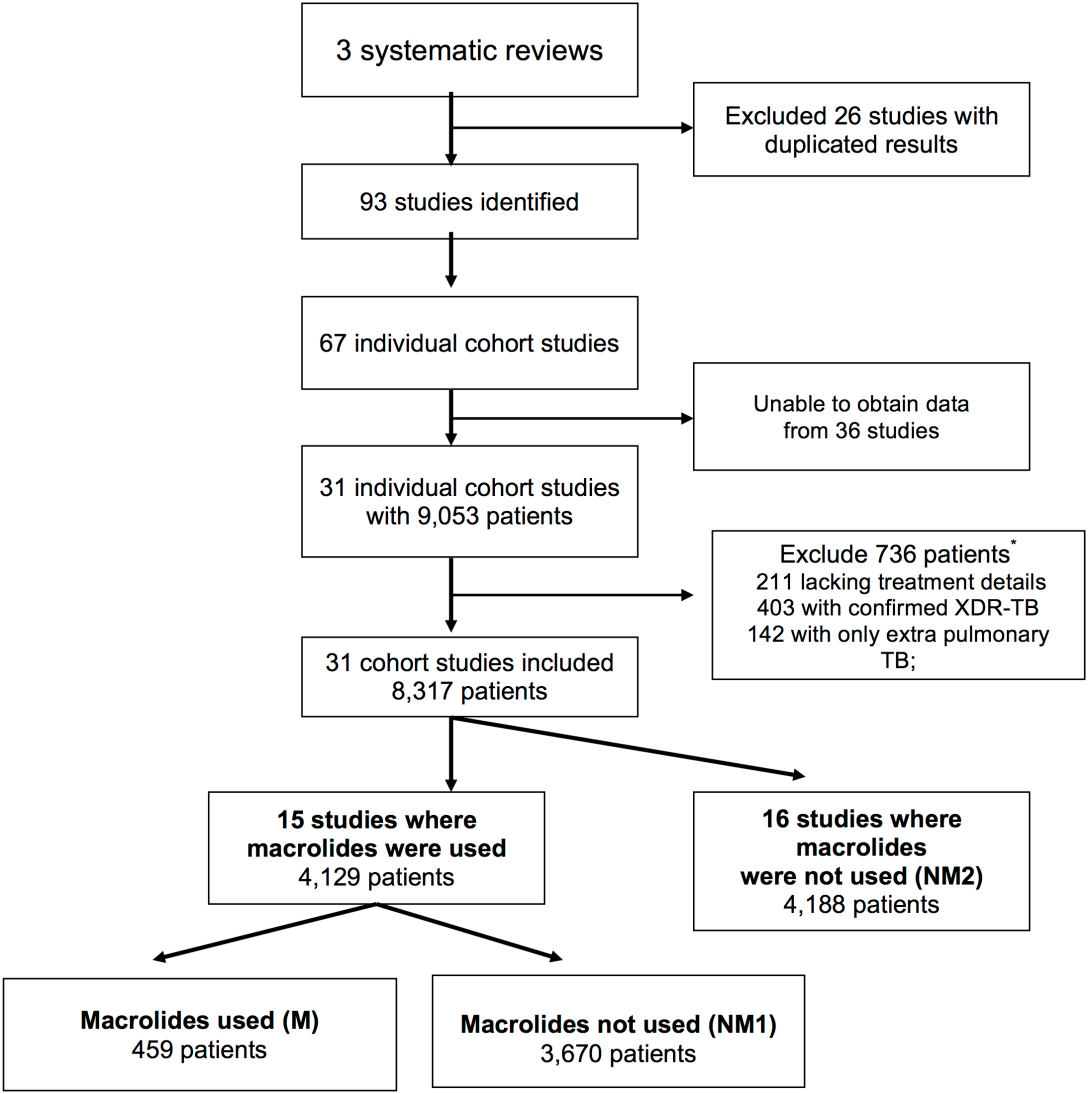
Consort diagram for an individual patient data meta-analysis of the effectiveness of macrolide antibiotics to treat multi-drug resistant tuberculosis. Group allocation was independent of which other antibiotics were concurrently used. *Some patients were excluded on the basis of more than one criterion.

Study methods are reported according to PRISMA criteria (S1 Table). Data for this individual patient data meta-analysis were collected from 31 observational studies, published after 1970, selected from three prior meta-analyses evaluating MDR-TB treatment, in preparation for an expert committee revising the World Health Organization (WHO) MDR-TB treatment guidelines (Figs 1 and 2) [19–21]. The method of contacting authors, collecting and extracting data, individual study characteristics and outcomes for each individual study have been reported [19]. Data were evaluated completeness, and additional information sought if required. Additional criteria for including studies were: the study authors could be contacted and were willing to share their data, the cohort included at least 25 subjects and the outcome of treatment success was reported. All included studies were retrospective or prospective observational studies, and therefore of low quality. The quality of included studies is summarized in S2 Table. No problems with data integrity were identified. Only patients with microbiologically confirmed MDR-TB were included. Individual patients with extensively drug resistant tuberculosis (XDR-TB, defined as MDR-TB plus resistance to any FQN and at least one second-line injectable antibiotic), only extrapulmonary TB or missing treatment information were excluded. Individual subject data included demographic characteristics, HIV status, the extent of disease, history of prior treatment, phenotypic drug susceptibility testing and treatment regimen. Extensive disease was defined as pulmonary TB with positive sputum smear and / or cavitation on chest radiograph. Studies where the selected antibiotic class was not used were excluded from the analyses of that class. For FQNs the comparison was between group F and group NF1 (Fig 1), and for macrolides between group M and group NM1 (Fig 2).

The main analyses assessed the association of either FQN antibiotics or macrolide antibiotics with treatment success (cure or treatment completion) against a combined primary outcome of treatment failure, relapse or death. A secondary comparison was success vs. fail or relapse (without death) for each drug class. Loss to follow-up was excluded as an outcome from both comparisons. Patients taking the antibiotic of interest were described as ‘exposed’, and those not taking the antibiotic as ‘unexposed’.

### Description of the Exposure

We hypothesized that after adequately controlling for confounding, the adjusted effect estimates should show a positive association between FQN use and the primary outcome, while there should be no evidence of an association for macrolide antibiotics.

#### Fluoroquinolone antibiotics

Until recently, later generation (newer) FQNs were substantially more expensive and may have been reserved for patients with more severe disease [22, 23] or those failing first-line MDR-TB therapy [24]. Consequently, confounding by indication may attenuate the apparent effectiveness of later generation FQNs. For this reason, separate analyses of the two classes of FQN antibiotics were performed, comprising either (a) later generation FQN antibiotics (including moxifloxacin, gatifloxacin, levofloxacin) against no FQN use, or (b) earlier generation FQN antibiotic (ofloxacin) against no FQN use. Patients taking ciprofloxacin (an earlier generation FQN) were excluded from the analyses, since it is considered largely ineffective in the clinical treatment of TB [21, 25], having only a minimal effect *in vitro* [26], and hence no longer recommended [17].

#### Macrolide antibiotics

The separate comparison was performed between a separate class of antibiotics, macrolides, and those taking no macrolides. As highlighted above, we did not expect macrolides to truly make patients better. At worst they are clinically ineffective, and not harmful, given that the drug class has an excellent safety record. Hence, there is no reason *a priori* to expect patient outcomes to be adversely affected by this class of antibiotics. Treatment outcomes with and without macrolide antibiotics were examined independently of whether FQN antibiotics were also used.

### Analytical Approaches

We evaluated different methods to control confounding for each of the three antibiotic groups (earlier generation FQNs, later generation FQNs or macrolides), and treatment success for MDR-TB [21]. Effects were estimated using generalized linear mixed models (GLMMs) (i.e. random effects logistic regression models) estimated via adaptive quadrature (QUAD).

**Fig 3:**
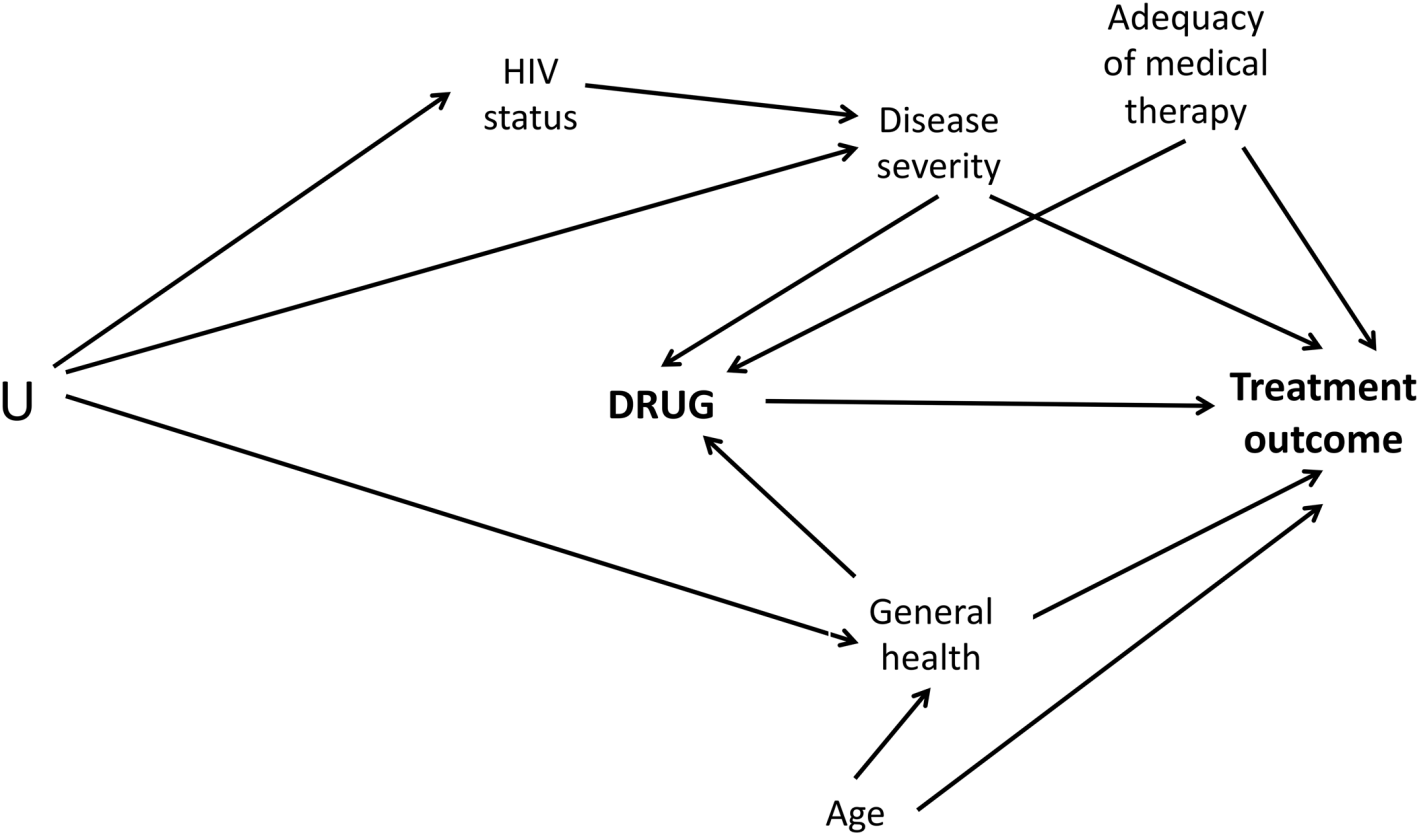
Directed Acyclic Graph describing the covariates affecting treatment outcomes for multi-drug resistant tuberculosis. Legend: U = Unmeasured confounder; HIV = Human immunodeficiency virus.

To identify the most important potential confounders in our dataset, we constructed a Directed Acyclic Graph (Fig 3). Analytic methods used to account for confounding included traditional multivariable methods and four different methods of adjustment using the propensity score: regression adjustment for propensity score quintiles or for the propensity score, propensity score-based matching and inverse probability of treatment weighting (IPTW).

In contrast to regression-based models, which give a conditional (unit specific) effect estimate, propensity score based models can estimate the marginal (population-average) effect of an intervention [27]. A propensity score is the probability of allocation to treatment given the measured covariates (6). If the dichotomous outcome variable Z represents treatment, and ***X*** is the vector of available baseline pre-treatment covariates, then the propensity score is defined as the conditional probability of being treated, given the measured covariates Pr(Z = 1 | ***X***). Propensity scores for each individual were estimated using logistic regression (Proc Logistic, SAS v9.3) (21), including the covariates that were available across all datasets. (22) These included baseline clinical characteristics (age, extent of disease, prior TB history, prior MDR-TB history and HIV status) and treatment factors (total duration of therapy and number of drugs in the intensive phase). Propensity score model specification was evaluated based on the comparison of covariates between matched subjects.

To estimate the associations between the antibiotics of interest and the outcomes, the following analytic approaches were taken:

i) Crude analysis
  An unadjusted mixed logistic regression was estimated, allowing intercepts and the effect of the antibiotic to vary across studies.
ii) Multivariable logistic regression
  A multivariable mixed logistic regression was performed including covariates chosen to account for likely causal relationships between potential confounders, with study and the effect of the antibiotic as random effects.
iii) Conditional analysis using propensity score
  The conditional propensity score model applied a GLMM with propensity score as a continuous variable, with study and the effect of the antibiotic as a random effect.
iv) Stratification by propensity score quintile
  Patients were ranked according to propensity score, and stratified into propensity score quintiles, an approach that has been estimated to remove 90% of bias in measured covariates [28]. Stratified analysis was performed using a GLMM with study and the effect of the antibiotic as a random effect.
v) Inverse probability of treatment weighting using propensity score
  In an inverse probability of treatment weighted (IPTW) model, individual observations in the regression were weighted by the reciprocal of the predicted probability of being in the treatment group (derived from the propensity score), normalized to the sample mean. Analysis was performed using a GLMM with study and the effect of the antibiotic as random effect, using normalized weights based upon the propensity score, adjusting for chosen covariates to account for potential confounders.
vi) Matching within and vii) matching across studies using propensity score
  Patients receiving the drug of interest (exposed) were matched with patients that did not receive this drug (unexposed) according to their propensity scores [29]. Two matching approaches were explored: 1) matching was restricted to within individual studies; and 2) matches to could be made across all studies, with the propensity score being calculated within or across studies, respectively. Matching was performed using three alternate approaches: one-to-one matching without replacement, one-to-one matching with replacement or (up to) four-to-one matching.

Each matching algorithm used nearest neighbor matching to identify pairs of treated and untreated subjects [30], whose propensity scores differed by no more than a specified amount (the caliper distance) which was fixed at 0.2 times the standard deviation of the logit of propensity score [31]. For matching with replacement, exposed subjects matched to a specified patient were returned to the pool of unexposed subjects and were eligible for subsequent selection. Treatment outcomes between groups were evaluated using a GLMM, with study, treatment and match as random effects, propensity score as a model covariate [32].

Optimal matching was defined as the strategy that achieved the closest covariate balance. This was calculated as the standardized difference of the covariates and of the logit of the propensity score between exposed and unexposed groups [33, 34], defined according to published methods [7].

Small differences in the average values of covariates between intervention and control groups after propensity score matching (<10%) implied an adequate balance had been achieved between the measured characteristics of matched subjects [35].

When a model did not converge, effects were estimated via penalized quasi likelihood (Proc Glimmix, SAS v9.3) [36]. Patients were clustered at the level of the study and intercepts and slopes of estimates of effect were allowed to vary across studies. The precision and size of effect estimates for FQNs and macrolides, expressed as odds ratios with 95% confidence intervals, were compared. We assumed missing observations were missing at random, with missing values of included covariates imputed using Markov Chain Monte Carlo methods.

### Meta-Analysis of Outcomes

A random effects meta-analysis of outcomes for each antibiotic was performed using the Der Simonean Laird method, with heterogeneity of intervention effect estimates evaluated using the I^2^ statistic and associated 95% confidence intervals [37].

In all models, if a hierarchical model could not be fitted, a fixed effects model was used. All analyses were performed in SAS, version 9.3 (SAS Institute, Cary, NC).

## Results

### Description of Study Population

FQN antibiotics were used by one patient or more in 28 of the 31 observational studies for which data was available (Fig 1). Among these studies, 6,612 patients received any FQN and 723 did not. Among those taking quinolones, 6,007 (90.8%) received an earlier generation FQN and 748 (11.3%) received a later generation FQN, including 148 who received both classes of drugs. As shown in Table 1, patients taking FQNs were slightly younger, less likely to have disease with lung cavitation, had lower rates of advanced drug resistance, and received a longer total duration of therapy.

**Table 1:** Characteristics of study participants, according to whether fluoroquinolone antibiotics were used.

	Fluoroquinolones used		Fluoroquinolones not used		
Variable	n	(%)	n	(%)	p-value
Total	6612		723		
Male gender	4546	(68.8%)	481	(66.5%)	0.221
Mean age, yrs (sd)	39.2	(13.5)	42.8	(15.4)	<0.001
**Extent of disease**					
Smear positive	4028	(73.3%)	481	(76.8%)	0.062
Extensive disease	4712	(72.6%)	520	(74.2%)	0.396
Pulmonary TB only	6247	(100%)	689	(100%)	0.905
Bilateral disease	904	(71%)	141	(66.8%)	0.223
Cavitary disease	3507	(66.9%)	261	(57.9%)	<0.001
**Antibiotic therapy**					
*Number of drugs (median*, *iqr)*					
In intensive phase	5	(5, 6)	4	(0, 5)	<0.001
In continuation phase	4	(3, 5)	3	(0, 4)	<0.001
*Duration of therapy (median*, *iqr)*					
Months total therapy	18	(16, 19)	9	(1, 19)	<0.001
Months intensive phase	3	(3, 6)	6	(3, 7)	<0.001
Months continuation phase	15	(8, 15)	0.2	(0, 5)	<0.001
**Degree of antibiotic resistance**					
MDR-TB only	4288	(76.7%)	253	(60.4%)	<0.001
MDR-TB+ FQN resistance	297	(5.3%)	110	(26.3%)	<0.001
MDR-TB + injectable resistance	1005	(18%)	56	(13.4%)	0.017
**Quinolone antibiotic used**					
Early generation quinolone^+^	6007	(90.8%)	—		—
Later generation quinolone***	748	(11.3%)	—		—

*XDR-TB is defined as MDR-TB with additional resistance to a FQN and an injectable antibiotic. The denominators used to calculate percentages differ slightly between each variable, in light of missing values.

**Some patients used more than one FQN class.

^+^Participants taking ciprofloxacin were excluded.

*** Later generation quinolones include levofloxacin, moxifloxacin and gatifloxacin. iqr = interquartile range

Macrolides were used in 15 included studies, within which 459 patients received macrolides and 3,670 did not (Fig 2).

### Outcomes of FQN Treatment

Table 2 shows the pooled estimates of FQN treatment outcome, stratified by FQN generation. Treatment success was similar regardless of whether an earlier or a later generation FQN was used. Default rates were lower among patients given later generation FQNs, when compared to those who did not receive FQNs. Mortality among those who did not take FQNs was higher than for than those taking FQNs.

**Table 2:** Pooled treatment outcomes by fluoroquinolone use, with pooled Der Simonian and Laird random effects estimates.

Group	Events	n	(%)	(95% CI)	I^2^ (%)	(95% CI I^2^)	τ^2^
**Treatment success**							
No quinolones	208	723	51%	(40, 63%)	95%	(93, 96%)	0.065
Earlier generation FQNs	3282	6007	61%	(53, 68%)	97%	(96, 97%)	0.032
Later generation FQNs	504	748	69%	(60, 79%)	88%	(82, 92%)	0.028
**Treatment failure or relapse**							
No quinolones	51	723	3%	(0, 6%)	59%	(35, 74%)	0.004
Earlier generation FQNs	487	6007	3%	(2, 4%)	40%	(4, 62%)	0.000
Later generation FQNs	79	748	7%	(3, 11%)	74%	(56, 84%)	0.004
**Default**							
No quinolones	239	723	21%	(11, 31%)	89%	(84, 92%)	0.048
Earlier generation FQNs	1505	6007	19%	(13, 24%)	96%	(95, 97%)	0.020
Later generation FQNs	97	748	10%	(5, 16%)	82%	(72, 89%)	0.009
**Death**							
No quinolones	225	723	17%	(3, 30%)	96%	(95, 97%)	0.102
Earlier generation FQNs	733	6007	9%	(6, 12%)	94%	(92, 95%)	0.005
Later generation FQNs	68	748	5%	(2, 8%)	65%	(39, 80%)	0.003

Patients taking ciprofloxacin were excluded from the analysis, Some patients received both earlier and a later generation fluoroquinolone antibiotic, in which case they were included in both analyses. CI = Confidence Interval. FQN = fluoroquinolone.

### Results of Different Analytic Methods to Adjust for Confounding

#### Crude and multivariable analyses

Crude (unadjusted) estimate showed that both early generation FQNs (odds ratio (OR) 3.2, 95% CI: 2.5, 4.2) and later generation FQNs (OR 3.2, 95% CI: 2.1, 5.0) were associated with treatment success. Multivariable analysis showed a similar effect for earlier and later generation FQNs.

#### Regression analysis using propensity score as a covariate

Analysis using propensity score quintile gave similar effect estimates as multivariable analysis. Regression with propensity score as a continuous covariate produced slightly smaller effect estimates than with propensity score quintiles.

#### Propensity score matching

Propensity scores could not be calculated within studies where outcomes or treatment allocation was homogenous (that is, the same outcome occurred in all patients in that study). Hence, 18 studies could be included for FQN analyses with matching within studies, and 15 studies included for macrolide analyses with matching within studies. Optimal covariate matching was achieved using one-to-one matching of subjects for propensity scores allocated across studies with replacement, based upon standardized difference for the propensity score and for individual covariates. Using this method, the standardized differences between the logit of the propensity scores between treated and untreated groups was less than 1% for all antibiotics (Table 3). The covariate balance achieved with each matching technique is shown in Tables 4, 5 and 6.

**Table 3:** Comparison of covariate balance before and after one to one matching across studies, with or without replacement, for each drug group, based upon standardized difference (%) of the logit of propensity score between groups.

			Matching within studies				Matching across studies			
Exposure	Before matching		Matching without replacement		Matching with replacement		Matching without replacement		Matching with replacement	
	Std dif ^#^ (%)	p-value^+^	Std dif (%)	p-value^+^	Std dif (%)	p-value	Std dif (%)	p-value^+^	Std dif (%)	p-value^+^
Earlier generation FQN	-18.74	<0.0001	-23.58	<0.001	-2.90	<0.001	-14.61	<0.001	**0.05**	**0.022**
Later generation FQN	-13.35	<0.0001	-26.53	<0.001	-17.44	<0.001	-12.79	<0.001	**0.13**	**0.14**
Macrolide	-26.45	<0.0001	12.48	0.06	9.01	<0.001	-6.37	0.118	**-0.03**	**0.542**

^#^ Std dif = Standardized difference of logit of propensity score in unexposed relative to exposed. FQN = fluoroquinolone.

^+^The differences in propensity score between exposed and unexposed individuals receiving were tested for significance using a paired t-test [34]

**Table 4:** Balancing achieved by different propensity score-based matching strategies for earlier generation fluoroquinolone use.

Covariate	Ofloxacin	No ofloxacin	Before matching		Matching within studyes, without replacement*		Matching within studies, with replacement*		Matching across studies without replacement*		Matching across studies with replacement*	
	Mean (sd)	Mean (sd)	Std dif ^#^ (%)	p-value^+^	Std dif (%)	p-value^+^	Std dif (%)	p-value	Std dif (%)	p-value^+^	Std dif (%)	p-value^+^
Age	39.06 (13.25)	40.91 (15.44)	4.54	0.0004	1.81	0.555	-7.54	<0.001	4.46	0.102	5.33	<0.001
Male gender	0.69 (0.46)	0.63 (0.48)	12.1	0.001	-2.43	0.4367	-10.45	<.0001	-1.70	0.5439	-2.51	0.0005
Extensive disease	0.72 (0.45)	0.8 (0.4)	-18	0	0.00	1.0000	1.54	0.1095	3.34	0.2172	3.29	<.0001
Prior TB	0.74 (0.44)	0.47 (0.5)	57.4	0	-3.84	0.1742	-8.53	<.0001	-6.69	0.0148	-1.87	0.0077
Prior MDR-TB	0.07 (0.26)	0.15 (0.36)	-24.3	0	4.59	0.1390	17.56	<.0001	4.22	0.1275	3.79	<.0001
Known HIV co-infection	0.15 (0.36)	0.16 (0.37)	-2.7	0.442	3.29	0.1824	-0.63	0.3608	-9.03	0.0018	-9.61	<.0001
No. drugs (intensive)	5.36 (0.98)	4.47 (2.41)	-17.1	<0.0001	-14.64	<0.001	3.67	<0.001	-12.24	<0.001	3.71	<0.001
Total therapy (months)	17.7 (6.66)	18.96 (15.83)	3.69	0.0129	-9.08	0.002	2.21	0.012	-4.77	0.074	-2.17	0.002
**Logit of predicted PS**	**3.09 (0.91)**	**2.17 (2.29)**	**-18.74**	**<0.0001**	**-23.58**	**<0.001**	**-2.90**	**<0.001**	**-14.61**	**<0.001**	**0.05**	**0.022**

* 1:1 matching.

^#^ Std Diff = Standardized difference for unexposed relative to exposed. The differences in characteristics between exposed and unexposed individuals receiving were tested for significance using a paired t-test for continuous variables and McNemar’s test for dichotomous variables [34]. PS = Propensity score. FQN = fluoroquinolone. MDR-TB = multi-drug resistant tuberculosis. TB = tuberculosis. HIV = human immunodeficiency virus.

**Table 5:** Balancing achieved by different propensity score-based matching strategies for later generation fluoroquinolone use.

Covariate	Later generation FQN	No later generation FQN	Before matching		Matching within studyes, without replacement*		Matching within studies, with replacement*		Matching across studies without replacement*		Matching across studies with replacement*	
	Mean (sd)	Mean (sd)	Std dif ^#^ (%)	p-value^+^	Std dif (%)	p-value^+^	Std dif (%)	p-value	Std dif (%)	p-value^+^	Std dif (%)	p-value^+^
Age	37.71 (14.75)	39.63 (13.53)	4.79	0.0017*	10.50	0.104	3.35	0.123	2.24	0.736	2.83	0.147
Male gender	0.59 (0.49)	0.69 (0.46)	-20.7	0	3.20	0.6698	13.50	<.0001	-2.84	0.6949	3.46	0.0817
Extensive disease	0.8 (0.4)	0.72 (0.45)	18.2	0	1.76	0.7815	1.31	0.5529	0.00	1.0000	-3.79	0.0480
Prior TB	0.53 (0.5)	0.71 (0.45)	-38.4	0	-1.52	0.7815	2.20	0.3508	-8.49	0.2008	13.55	<.0001
Prior MDR-TB	0.23 (0.42)	0.07 (0.26)	45.3	0	4.90	0.3173	-0.99	0.6858	-5.37	0.4054	-11.14	<.0001
Known HIV co-infection	0.02 (0.15)	0.17 (0.38)	-51.5	0	6.18	0.1573	3.44	0.0578	22.64	0.0039	8.28	<.0001
No. drugs (intensive)	5.73 (1.58)	5.13 (1.36)	-14.37	<0.0001	1.84	0.746	2.65	0.226	-10.53	<0.001	0.54	0.453
Total therapy (months)	26.07 (16.2)	16.84 (6.94)	-26.2	<0.0001	-6.86	0.053	-6.52	<0.001	-14.70	0.016	-16.61	<0.001
**Logit of predicted propensity score**	**3.39 (1.48)**	**2.86 (1.29)**	-**13.35**	**<0.0001**	**-26.53**	**<0.001**	**-17.44**	**<0.001**	**-12.79**	**<0.001**	**0.13**	**0.14**

*One to one matching.

^#^ Std dif = standardized difference for unexposed relative to exposed. FQN = fluoroquinolone. The differences in characteristics between exposed and unexposed individuals receiving were tested for significance using a paired t-test for continuous variables and McNemar’s test for dichotomous variables [34]. MDR-TB = multi-drug resistant tuberculosis. TB = tuberculosis. HIV = human immunodeficiency virus.

**Table 6:** Balancing achieved by different propensity score-based matching strategies for macrolide use.

Covariate	Macrolide	No macrolide	Before matching		Matching within studyes, without replacement*		Matching within studies, with replacement*		Matching across studies without replacement*		Matching across studies with replacement*	
	Mean (sd)	Mean (sd)	Std dif ^#^ (%)	p-value^+^	Std dif (%)	p-value^+^	Std dif (%)	p-value	Std dif (%)	p-value^+^	Std dif (%)	p-value^+^
Age	38.75 (13.11)	39.13 (13.45)	1.02	0.6192	-3.57	0.628	-8.11	<0.001	-7.28	0.31	-7.68	<0.001
Male gender	0.64 (0.48)	0.68 (0.47)	-8.7	0.127	0.00	1.0000	8.64	<.0001	-1.56	0.8273	4.72	0.0021
Extensive disease	0.79 (0.41)	0.72 (0.45)	15.8	0.008	6.64	0.4054	-3.93	0.0174	-18.03	0.0116	-0.42	0.7973
Prior TB	0.57 (0.5)	0.7 (0.46)	-26	0	7.40	0.3458	-11.72	<.0001	7.72	0.2752	16.47	<.0001
Prior MDR-TB	0.26 (0.44)	0.08 (0.27)	47.8	0	-11.00	0.1573	-2.44	0.1159	-7.87	0.2850	-9.11	<.0001
Known HIV co-infection	0.04 (0.19)	0.17 (0.38)	-44.7	0	-4.73	0.3173	0.24	0.7389	18.08	0.0196	6.29	0.0002
No. drugs (intensive)	6.44 (1.69)	5.12 (1.33)	-30.55	<0.0001	-12.91	0.059	-31.10	<0.001	-10.03	0.028	-5.89	<0.001
Total therapy (months)	24.38 (16.32)	17.55 (8.28)	-18.68	<0.0001	-3.49	0.337	-7.70	0.337	-14.69	0.04	-5.53	<0.001
**Logit of predicted propensity score**	**3.95 (1.6)**	**2.86 (1.28)**	**-26.45**	**<0.0001**	**12.48**	**0.06**	**9.01**	**<0.001**	-6.37	0.118	**-0.03**	**0.542**

*1:1 matching.

^#^ Std dif = Standardized difference for unexposed relative to exposed. The differences in characteristics between exposed and unexposed individuals receiving were tested for significance using a paired t-test for continuous variables and McNemar’s test for dichotomous variables [34].

MDR-TB = multi-drug resistant tuberculosis. TB = tuberculosis. HIV = human immunodeficiency virus.

#### Inverse probability of treatment weighting

IPTW analysis yielded marginal effect estimates that were non-significant for earlier generation FQNs, but significant for later generation FQNs (OR 4.0, 95 CI: 1.5, 10.5).

### Outcomes of Macrolide Antibiotic Treatment

Applying the same analytic strategies to macrolide antibiotics, the odds ratio was consistently less than one for all analytic methods, as shown in Table 2. The effect estimate was closest to the null using the method of propensity score matching across studies with replacement (OR 0.7, 95% CI: 0.4, 1.1).

Table 7 compares the effect estimates produced using each analytic approach. Matching within studies produced generally smaller effect estimates than other multivariable approaches. Matching with replacement resulted in effect estimates that were closer to the null for matched analyses of FQN antibiotics (with propensity score allocated within or across studies) than for multivariable approaches. This was also observed for macrolide antibiotics, when matching within macrolide studies. However, for matching across macrolide studies, there was no substantial change in effect estimate when replacement was used. Stratification by propensity score quintiles found that odds ratios were similar across strata (Table 8).

**Table 7:** The relationship between antibiotic use and successful treatment of tuberculosis (versus death, relapse or failure), applying different analytic methods to account for confounding.

Method of adjustment	Early generation FQN versus no FQN ^a^		Later generation FQN versus no FQN ^a^		Macrolide antibiotics ^b^	
	OR	95% CI	OR	95% CI	OR	95% CI
**Conventional approaches**						
Unadjusted estimate	**3.2**	**(2.5, 4.2)**	**3.2**	**(2.1, 5.0)**	**0.5**	**(0.4, 0.6)**
Multivariable analysis	**2.3**	**(1.7, 3.2)**	**3.2**	**(2.0, 5.2)**	**0.4**	**(0.3, 0.6)**
**Unmatched Propensity Score-based methods**						
Regression with PS quintile as covariate	**2.7**	**(2.1, 3.6)**	**3.3**	**(2.1, 5.2)**	**0.5**	**(0.4, 0.6)**
Regression with continuous PS as covariate	**2.0**	**(1.5, 2.7)**	**2.3**	**(1.5, 3.6)**	**0.5**	**(0.4, 0.6)**
**Propensity Score matching within studies**						
1:1 matching^¶^ no replacement	**1.9**	**(1.1, 3.3)**	0.9	(0.2, 4.8)	**0.5**	**(0.4, 0.8)**
1:1 matching with replacement	0.9	(0.3, 2.7)	0.8	(0.2, 3.6)	0.7	(0.4, 1.1)
1:4 matching^Δ^	na		na		**0.5**	**(0.3, 0.7)**
**Propensity Score matching across studies**						
1:1 matching^+^ no replacement	**2.4**	**(1.3, 4.3)**	**2.5**	**(1.4, 4.3)**	**0.5**	**(0.3, 0.8)**
1:1 matching^¶^ with replacement	1.4	(0.5, 3.9)	1.5	(0.4, 5.4)	**0.5**	**(0.3, 0.8)**
1:4 matching^Δ^	na		na		**0.5**	**(0.4, 0.7)**
Inverse probability of treatment weighting based on propensity score	1.9	(0.8, 4.7)	**4.0**	**(1.5, 10.5)**	**0.5**	**(0.3, 0.9)**

The comparisons presented in this table exclude individuals lost to follow-up.

^**a**^ Comparison between those taking fluoroquinolones and those not taking fluoroquinolones, in studies where fluoroquinolones were used.

^b^ Comparison between those taking macrolides and those not taking macrolides, in studies where macrolides were used.

^**¶**^ Propensity scores calculated within individual studies. Ten studies were excluded owing to insufficient numbers of patients to perform analysis.

^+^ Matching by propensity score calculated across all studies. PS = Propensity score. OR = odds ratios. 95% CI = 95% confidence intervals. Bolded text indicates p <0.05. Macrolides includes azithromycin, clarithromycin and roxithromycin.

^**Δ**^ Up to four unexposed subjects for each subject taking active treatment. This analysis was only performed for macrolides, as a large number of unexposed subjects were available. na = not applicable, as insufficient unexposed subjects available to perform four to one matching.

**Table 8:** The relationship between antibiotic use and successful treatment of tuberculosis (versus death or failure—excluding relapse and loss to follow-up) applying different analytic methods to account for confounding.

Method of adjustment	Earlier generation FQN ^a^		Later generation FQNs ^a^		Macrolide antibiotics ^b^	
	OR	95% CI	OR	95% CI	OR	95% CI
**Conventional approaches**						
Unadjusted estimate	**2.3**	**(1.5, 3.3)**	**1.5**	**(0.9, 2.7)**	**0.3**	**(0.2, 0.5)**
Multivariable analysis	**2.1**	**(1.4, 3.2)**	**2.0**	**(1.1, 3.6)**	**0.3**	**(0.2, 0.5)**
**Unmatched PS based methods**						
Regression with PS quintile as covariate	**2.1**	**(1.4, 3.0)**	**1.8**	**(1.0, 3.1)**	**0.3**	**(0.2, 0.5)**
Regression with continuous PS as covariate	**1.9**	**(1.2, 2.8)**	**1.5**	**(0.9, 2.7)**	**0.4**	**(0.3, 0.5)**
**Inverse probability of treatment weighting**	1.8	(0.7–4.3)	1.1	(0.2, 5.4)	**0.4**	**(0.3, 0.6)**
PS matching within studies						
1:1 matching,^¶^ no replacement	1.5	(0.5, 4.3)	0.7	(0.1, 4.8)	0.5	(0.3, 0.9)
1:1 matching with replacement	0.5	(0.1, 2.2)	0.2	(0.0, 2.1)	0.5	(0.3, 0.8)
1:4 matching^**Δ**^	na		na		0.4	(0.3, 0.7)
PS matching across studies						
1:1 matching^+^ no replacement	2.4	(0.8, 7.6)	2.0	(0.3, 12.3)	0.4	(0.2, 0.7)
1:1 matching^¶^ with replacement	0.5	(0.1, 2.2)	0.4	(0.1, 1.9)	0.4	(0.2, 0.8)
1:4 matching^**Δ**^	na		na		0.4	(0.2, 0.6)

^**a**^ Comparison between those taking fluoroquinolones and those not taking fluoroquinolones, in studies where fluoroquinolones were used.

^b^ Comparison between those taking macrolides and those not taking macrolides, in studies where macrolides were used.

^**¶**^ Propensity scores calculated within individual studies. Ten studies were excluded owing to insufficient numbers of patients to perform analysis.

^+^ Matching by propensity score calculated across all studies. PS = Propensity score. OR = odds ratios. 95% CI = 95% confidence intervals. Bolded text indicates p <0.05. Macrolides includes azithromycin, clarithromycin and roxithromycin. na = not applicable, as insufficient unexposed subjects available to perform four to one matching. FQN = fluoroquinolone. These analyses excluded individuals with relapse or loss-to follow-up.

### Modeling of Secondary Outcomes

Using the secondary outcome treatment success versus treatment failure or relapse (excluding death and default, i.e. a smaller dataset), effect estimates were smaller and less precise, but in the same direction those obtained using the main comparison (success versus death, failure or relapse) (Table 8).

The numbers of individuals included from each study are shown in Table 9. Analyses for each generation of FQN, stratified according to propensity score quintiles, are shown in Table 10.

**Table 9:** Subjects by study, stratified by fluoroquinolone and macrolide use.

Study	Fluoroquinolone antibiotic used		Macrolide antibiotic used		Total
	n	(%)	n	(%)	n
Avendano	66	(93%)	8	(11%)	71
Burgos	24	(64.9%)	-	-	37
Chan	101	(71.1%)	12	(6.2%)	142
Chiang	122	(97.6%)	-		125
Cox	77	(100%)	-		77
Geerligs	35	(89.7%)	1	(2.3%)	39
Granich/Banerj	27	(31.8%)	-	-	85
Holtz	2174	(100%)	-	-	2174
Kim/Shim	1102	(85.6%)	25	(1.9%)	1288
Kim/Yim	156	(85.7%)	46	(25.3%)	182
Kwon	123	(96.1%)	33	(25.6%)	128
Leimane	926	(98%)	79	(8.4%)	945
Lockman	208	(95.4%)	30	(13.8%)	218
Masjedi	27	(100%)	-	-	27
Migliori	76	(91.6%)	13	(15.7%)	83
Mitnick	147	(98.7%)	103	(15.7%)	149
Munsiff/Li	131	(40.6%)	-		323
Narita	44	(67.7%)	2	(3%)	65
O’Riordan	16	(84.2%)	9	(32.1%)	19
Perez-Guzman	7	(43.8%)	19	(57.6%)	16
Palmero	78	(70.9%)	-	-	110
Park	131	(100%)	-	-	131
Schaaf	33	(91.7%)	-	-	36
Shin	477	(89.2%)	-	-	535
Shiraishi	51	(92.7%)	-	-	55
Tupasi	121	(89.6%)	75	(49%)	135
Uffredi	33	(80.5%)	4	(9.8%)	41
Yew	99	(100%)	-	-	99
**Total**	**6612**		**459**		**7335**

**Table 10:** Stratification by propensity score quintiles, using propensity score as a model covariate.

Stratum	OR	(95% CI)
**Early generation FQN vs. no FQN**^a^		
Stratum 1	2.3	(1.4, 3.7)
Stratum 2	1.3	(0.5, 3.4)
Stratum 3	1.3	(0.3, 5.2)
Stratum 4	2.3	(0.5, 11.2)
Stratum 5	3.2	(1.4, 7.5)
**Later generation FQN vs. no FQN**^a^		
Stratum 1	2.5	(1.0, 6.5)
Stratum 2	1.9	(0.5, 7.6)
Stratum 3	3.0	(0.4, 24.9)
Stratum 4	1.1	(0.2, 7.3)
Stratum 5	1.7	(0.8, 3.9)

^**a**^ Comparison between those taking fluoroquinolones and those not taking fluoroquinolones, in studies where fluoroquinolones were used.

FQN = Fluoroquinolones. OR = odds ratio. CI = confidence intervals.

The overall quality assessment for the primary and secondary measures was ‘very low’ according to GRADE criteria [38], on account of moderate to serious inconsistency between study outcomes and a serious risk of bias as all included data were from observational studies (S2 Table). A total of 6.3% of covariate values were imputed for the fluoroquinolone analyses, and 6.1% of covariate values were imputed for the macrolide analyses.

## Discussion

Meta-analysis of IPD from multiple observational studies can provide the best available source of evidence for an intervention, in the absence of randomized controlled trials, if the included data are representative of all available studies. However, analytic approaches must appropriately address bias due to confounding. In our meta-analysis of 31 observational studies of antibiotic therapy for MDR-TB, we accounted for confounding using a number of different analytic strategies that incorporated individual patient covariates, for two different drug classes. Propensity score matching achieved adequate covariate balance between exposed and unexposed individuals—the closest when one to one propensity score matching with replacement was used. Other propensity-score based approaches yielded more conservative effect estimates than using a traditional multivariable approach.

The importance of confounding by indication in observational studies is well-recognized [7]. However, it is often difficult to determine the optimal adjustment approach without knowledge of the unbiased effect estimate (i.e. the ‘truth’). In our study, substantial differences were seen between the baseline covariates of individual patients in exposed and unexposed groups for both drug classes tested, prior to adjustment. This supports the importance of adjustment of confounding by indication in these studies. Most analytic approaches generally brought the effect estimate closer to the null, in comparison to the unadjusted analysis.

Our findings are consistent with the presence of confounding by indication, an important source of bias which must be considered when interpreting observational studies. Furthermore, the propensity score-based methods gave outcomes that were broadly consistent with laboratory studies and published randomized trials of FQNs in drug susceptible TB. Our findings also aligned with current consensus guidelines for the treatment of both FQNs and macrolides.

Our findings do not support our initial hypothesis that later generation FQNs would be given more often in more advanced disease. The degree of improvement in covariate balance achieved using propensity score matching, as represented by the logit of the predicted propensity score, was similar for both classes of FQN antibiotics. However, analysis of early generation FQNs produced consistently lower estimates of effect than later generation FQNs, in line with expert opinion, and clinical guidelines [39]. Confidence intervals (CIs) for new generation FQNs were wider than for early generation FQNs, on account of the smaller number of patients taking this treatment.

For those taking macrolide antibiotics, which are considered to be safe clinically, yet ineffective in the laboratory setting, treatment outcomes were consistently worse for exposed than for unexposed individuals. This would be consistent with confounding by indication, since we do not expect macrolides—a widely used class of drug that is well-tolerated—to truly worsen outcomes. Traditional multivariable approaches did not fully correct for this potential bias. We found that propensity score methods did improve covariate balance, bringing the effect estimate closer to the null. Residual confounding is a more likely explanation than drug toxicity, in light of the favorable safety profile of macrolide antibiotics [40], however the latter cannot be excluded. This suggests that while propensity score matching can correct for some confounding by indication, interpretation of the outcome must allow for possible residual bias, if confounding by indication is substantial.

This study has potential clinical implications. First, our findings indicate that macrolides have limited benefit treatment of MDR-TB. This is in keeping with current WHO guidelines that assign the drugs to ‘Group 5’–i.e. those drugs of unproven effectiveness [9]. Second, most analytic approaches yielded a similar benefit for both early and late generation FQNs. This suggests that ofloxacin should be retained as an effective treatment option for MDR-TB patients. Furthermore, a high mortality was seen among those without FQNs. Together, these findings support the continuing use of FQN antibiotics as a part of standard MDR-TB therapy.

A potential limitation in this study was the absence of data for other clinically important covariates, which may influence treatment allocation or outcomes. While our models incorporated all relevant covariates for which data was available, it is possible other unmeasured confounders were unavailable in the combined individual patient dataset—leading to residual confounding [8]. Additionally, treatment group assignment was not allowed to vary over time in our model, despite the possibility that individualized regimens may be adjusted several times during the course of treatment. Additional confounding by indication by time-varying covariates may be introduced, as subsequent treatment decisions reflect changes in baseline characteristics. This limitation is challenging to overcome in pooled individual patient datasets, where only variables that are common to all studies can be analyzed. Of course, randomized studies would best address this concern, since on average balance will be achieved in measured and unmeasured characteristics. However, as no randomized trials of MDR-TB therapies have been published to date, IPD meta-analysis remains the best available source of data from which causal inference may be drawn.

We have applied a number of alternative statistical approaches to our dataset, however the ‘optimal’ statistical method remains unclear. Propensity score matching across studies with replacement achieved the closest covariate balance, leading to effect estimates that were closer to the null. This may indicate that residual confounding was of a greater magnitude when the other matching strategies were employed. However, as the true effect of the drugs is unknown this cannot be verified. A next step in evaluating the analytic approaches to IPD will be to perform simulation using precisely defined datasets with known bias. In particular, the effect of propensity-score based adjustment in the presence of varying degrees of unmeasured confounding needs to be explored further. Simulated datasets can be designed such that a ‘true’ treatment effect is known under a variety of conditions [41], allowing the effect of different approaches to be explored. In addition, application of these methods within the context of future randomized clinical studies, where unmeasured confounding is unlikely, may also be used to test how propensity score-based methods influence effect estimates in a clinical context.

## Conclusions

In this individual patient data meta-analysis, different methods of adjusting for individual patient covariates for observational studies yielded comparable results. Our analyses produced treatment effects that were generally reliable in direction and magnitude. This consistency is reassuring, and suggests that either multivariable or propensity score based methods could be used to account for measured confounding in this population. IPD meta-analysis offer considerable advantages, in accounting for confounding by indication more effectively than conventional ‘two step’ meta-analytic approaches. Wherever possible, a number of alternative methods to reduce bias should be used in evaluation of IPD meta-analysis of observational studies. However, when these methods are used, interpretation of adjusted estimates must still consider the potential for residual bias. Further simulation studies are warranted in order to explore the effect of residual confounding the estimates obtained with these analytic methods.

## Supporting Information

S1 Table PRISMA Check-list for individual patient data meta-analysis. (PDF)

S2 Table Study characteristics assessing the quality of the included studies. (DOCX)

## Abbreviations

aOR: adjusted odds ratio
CI: confidence intervals
FEV1: forced expiratory volume in 1 second
FQN: fluoroquinolone
GLMM: generalized linear mixed model
HIV: human immunodeficiency virus
IPD: individual patient data
IPTW: inverse probability weighting
MDR-TB: multi-drug resistant tuberculosis
OR: odds ratio
QUAD: quadrature
TB: tuberculosis
WHO: World Health Organization
XDR-TB: extensively drug resistant tuberculosis

## References

[1] Lambert PC, Sutton AJ, Abrams KR, Jones DR. A comparison of summary patient-level covariates in meta-regression with individual patient data meta-analysis. J Clin Epidemiol. 2002;55(1):86–94. . 11781126 10.1016/s0895-4356(01)00414-0

[2] Simmonds MC, Higgins JP, Stewart LA, Tierney JF, Clarke MJ, Thompson SG. Meta-analysis of individual patient data from randomized trials: a review of methods used in practice. Clin Trials. 2005;2(3):209–17. Epub 2005/11/11. . 16279144 10.1191/1740774505cn087oa

[3] Fibrinogen Studies C, Jackson D, White I, Kostis JB, Wilson AC, Folsom AR, et al Systematically missing confounders in individual participant data meta-analysis of observational cohort studies. Stat Med. 2009;28(8):1218–37. 10.1002/sim.3540 19222087 PMC2922684

[4] Thomas D, Radji S, Benedetti A. Systematic review of methods for individual patient data meta- analysis with binary outcomes. BMC Med Res Methodol. 2014;14:79 10.1186/1471-2288-14-79 24943877 PMC4074845

[5] Xu Z, Kalbfleisch JD. Propensity score matching in randomized clinical trials. Biometrics. 2010;66(3):813–23. 19995353 10.1111/j.1541-0420.2009.01364.x PMC3407414

[6] Rosenbaum P, Rubin D. The central role of the propensity score in observational studies for causal effects. Biometrika. 1983;70(1):41–55. 10.1093/biomet/70.1.41

[7] Austin PC. An Introduction to Propensity Score Methods for Reducing the Effects of Confounding in Observational Studies. Multivariate behavioral research. 2011;46(3):399–424. 10.1080/00273171.2011.568786 21818162 PMC3144483

[8] Austin PC, Grootendorst P, Anderson GM. A comparison of the ability of different propensity score models to balance measured variables between treated and untreated subjects: a Monte Carlo study. Stat Med. 2007;26(4):734–53. 16708349 10.1002/sim.2580

[9] World Health Organization. Treatment of Tuberculosis Guidelines—Fourth Edition Geneva: WHO 2010 Available: http://www.who.int/tb/publications/2010/9789241547833/en/. Accessed 1 December 2013.

[10] Mitnick CD, Castro KG, Harrington M, Sacks LV, Burman W. Randomized trials to optimize treatment of multidrug-resistant tuberculosis. PLoS Med. 2007;4(11):e292 10.1371/journal.pmed.0040292 17988168 PMC2062482

[11] Ziganshina LE, Titarenko AF, Davies GR. Fluoroquinolones for treating tuberculosis (presumed drug-sensitive). Cochrane DB Syst Rev. 2013;6:CD004795 Epub 2013/06/08. 10.1002/14651858.CD004795.pub4 . 23744519 PMC6532730

[12] Conde MB, Efron A, Loredo C, De Souza GR, Graca NP, Cezar MC, et al Moxifloxacin versus ethambutol in the initial treatment of tuberculosis: a double-blind, randomised, controlled phase II trial. Lancet. 2009;373(9670):1183–9. 10.1016/S0140-6736(09)60333-0 19345831 PMC2866651

[13] Pranger AD, Alffenaar JW, Aarnoutse RE. Fluoroquinolones, the cornerstone of treatment of drug-resistant tuberculosis: a pharmacokinetic and pharmacodynamic approach. Curr Pharm Des. 2011;17(27):2900–30. Epub 2011/08/13. . 21834759 10.2174/138161211797470200

[14] World Health Organization. Global tuberculosis report 2014. Geneva: WHO, 2014.

[15] Migliori GB, Zellweger JP, Abubakar I, Ibraim E, Caminero JA, De Vries G, et al European union standards for tuberculosis care. Eur Respir J. 2012;39(4):807–19. Epub 2012/04/03. 10.1183/09031936.00203811 . 22467723 PMC3393116

[16] American Thoracic Society / Centers for Disease Control and Pevention / Infectious Diseases Society of America. Treatment of Tuberculosis joint statement. Am J Respir Crit Care Med. 2003;167(4):603–62. 12588714 10.1164/rccm.167.4.603

[17] World Health Organization. Guidelines for the programmatic management of drug-resistant tuberculosis Geneva 2011. 23844450

[18] Truffot-Pernot C, Lounis N, Grosset JH, Ji B. Clarithromycin is inactive against *Mycobacterium tuberculosis*. Antimicrob Agents Ch. 1995;39(12):2827–8. 8593032 10.1128/aac.39.12.2827 PMC163042

[19] Ahuja SD, Ashkin D, Avendano M, Banerjee R, Bauer M, Bayona JN, et al Multidrug resistant pulmonary tuberculosis treatment regimens and patient outcomes: an individual patient data meta-analysis of 9,153 patients. PLoS Med. 2012;9(8):e1001300 10.1371/journal.pmed.1001300 22952439 PMC3429397

[20] Migliori GB, Sotgiu G, Gandhi NR, Falzon D, DeRiemer K, Centis R, et al Drug resistance beyond extensively drug-resistant tuberculosis: individual patient data meta-analysis. Eur Respir J. 2013;42(1):169–79. 10.1183/09031936.00136312 . 23060633 PMC4498806

[21] Falzon D, Jaramillo E, Schunemann HJ, Arentz M, Bauer M, Bayona J, et al WHO guidelines for the programmatic management of drug-resistant tuberculosis: 2011 update. Eur Respir J. 2011;38(3):516–28. 10.1183/09031936.00073611 . 21828024

[22] Gupta R, Kim JY, Espinal MA, Caudron JM, Pecoul B, Farmer PE, et al Public health. Responding to market failures in tuberculosis control. Science. 2001;293(5532):1049–51. 10.1126/science.1061861 . 11463877

[23] Drug patent watch. Details for generic name: levofloxacin [cited 2015 March 10]. Available: http://www.drugpatentwatch.com/ultimate/generic-api/levofloxacin. Accessed 23 March 2015.

[24] Cox H, Ford N, Keshavjee S, McDermid C, von Schoen-Angerer T, Mitnick C, et al Rational use of moxifloxacin for tuberculosis treatment. The Lancet Infect Dis. 2011;11(4):259–60. 10.1016/S1473-3099(11)70036-6 . 21453864

[25] Shandil RK, Jayaram R, Kaur P, Gaonkar S, Suresh BL, Mahesh BN, et al Moxifloxacin, ofloxacin, sparfloxacin, and ciprofloxacin against *Mycobacterium tuberculosis*: evaluation of in vitro and pharmacodynamic indices that best predict in vivo efficacy. Antimicrob Agents Ch. 2007;51(2):576–82. 10.1128/AAC.00414-06 17145798 PMC1797767

[26] Sirgel FA, Botha FJ, Parkin DP, Van de Wal BW, Schall R, Donald PR, et al The early bactericidal activity of ciprofloxacin in patients with pulmonary tuberculosis. Am J Respir Crit Care Med. 1997;156(3 Pt 1):901–5. 10.1164/ajrccm.156.3.9611066 . 9310011

[27] Loux TM, Drake C, Smith-Gagen J. A comparison of marginal odds ratio estimators. Statistical methods in medical research. 2014 10.1177/0962280214541995 . 25006032

[28] Rosenbaum PR, Rubin DB. Reducing bias in observational studies using subclassification on the propensity score. J am Stat Assoc. 1994;79(387):516–4.

[29] Rubin DB, Thomas N. Matching using estimated propensity scores: relating theory to practice. Biometrics. 1996;52(1):249–64. . 8934595

[30] Coca-Perraillon M, editor Matching with Propensity Scores to Reduce Bias in Observational Studies,. Proceedings of the NorthEast SAS Users Group Conference (NESUG); 2006; Philadelphia, PA.

[31] Austin PC. Optimal caliper widths for propensity-score matching when estimating differences in means and differences in proportions in observational studies. Pharmaceutical statistics. 2011;10(2):150–61. 10.1002/pst.433 20925139 PMC3120982

[32] SAS Institute. PROC MI: SAS Institute Inc; 2007. Available: http://www.sas.com. Accessed 18 June 2014.

[33] D'Agostino RB Jr. Propensity score methods for bias reduction in the comparison of a treatment to a non-randomized control group. Stat Med. 1998;17(19):2265–81. . 9802183 10.1002/(sici)1097-0258(19981015)17:19<2265::aid-sim918>3.0.co;2-b

[34] Austin PC. The performance of different propensity score methods for estimating marginal hazard ratios. Stat Med. 2013;32(16):2837–49. 10.1002/sim.5705 23239115 PMC3747460

[35] Normand ST, Landrum MB, Guadagnoli E, Ayanian JZ, Ryan TJ, Cleary PD, et al Validating recommendations for coronary angiography following acute myocardial infarction in the elderly: a matched analysis using propensity scores. J Clin Epidemiol. 2001;54(4):387–98. . 11297888 10.1016/s0895-4356(00)00321-8

[36] SAS Institute. PROC GLIMMIX: SAS Institute Inc; 2007. Available: http://www.sas.com. Accessed 18 June 2014.

[37] Higgins JP, Thompson SG. Quantifying heterogeneity in a meta-analysis. Stat Med. 2002;21(11):1539–58. 10.1002/sim.1186 . 12111919

[38] Guyatt G, Oxman AD, Akl EA, Kunz R, Vist G, Brozek J, et al GRADE guidelines: 1. Introduction-GRADE evidence profiles and summary of findings tables. J Clin Epidemiol. 2011;64(4):383–94. Epub 2011/01/05. 10.1016/j.jclinepi.2010.04.026 . 21195583

[39] World Health Organization. Guidelines for the programmatic management of drug-resistant tuberculosis—2011 update. Geneva: WHO 2011. 23844450

[40] Rubinstein E. Comparative safety of the different macrolides. Int J Antimicrob Ag. 2001;18 Suppl 1:S71–6. . 11574199 10.1016/s0924-8579(01)00397-1

[41] Burton A, Altman DG, Royston P, Holder RL. The design of simulation studies in medical statistics. Stat Med. 2006;25(24):4279–92. 10.1002/sim.2673 . 16947139

