# Supplementary material for: Propensity Score-Based Approaches to Confounding by Indication in Individual Patient Data Meta-Analysis: Non-Standardized Treatment for Multidrug Resistant Tuberculosis
Source: PLoS One. 2016 Mar 29;11(3):e0151724. doi: 10.1371/journal.pone.0151724 (PMC4811416; doi:10.1371/journal.pone.0151724)
Supplement: S2 Table — (DOCX) [file pone.0151724.s002.docx]

**S2 Table: Study characteristics assessing the quality of the included studies**

| **First author** | **Years of study** | **Location** | **Type of Drug Regimen (with second line drugs)** | **Allocation conceal-ment** | **Incomplete outcome data addressed** | **Blinding of participants, personnel and outcome assessors to primary outcome** | **Free of selective reporting** | **Risk of bias**** |
| --- | --- | --- | --- | --- | --- | --- | --- | --- |
| Burgos (Burgos) | 1983-2000 | USA (San Francisco) | Individualized | No | Yes | No | Yes | Serious |
| Chan (Strand) | 1984-1998 | USA (Colorado) | Individualized | No | Yes | No | Yes | Serious |
| Chiang (Enarson) | 1992-1996 | Taiwan (Taipei) | Individualized | No | Yes | No | Yes | Serious |
| Cox (Cox) | 2003-2005 | Uzbekistan | Individualized | No | Yes | No | Yes | Serious |
| De Riemer (Garcia-Garcia) | 1994-2009 | Mexico (Veracruz) | Standardized^@^ | No | Yes | No | Yes | Serious |
| Escudero (Pena) | 1998-2000 | Spain (Madrid) | Individualized | No | Yes | No | Yes | Serious |
| Geerligs (van der Werf) | 1987-1988, 1998-2008 | The Netherlands | Individualized | No | Yes | No | Yes | Serious |
| Holtz (Van der Walt) | 2000-2004 | South Africa  (All centres) | Standardized | No | Yes | No | Yes | Serious |
| DH Kim (Shim) | 2000-2002 | South Korea  (Seoul) | Individualized | No | Yes | No | Yes | Serious |
| HR Kim (Yim) | 1980-2007 | South Korea  (Seoul) | Individualized | No | Yes | No | Yes | Serious |
| Kwon (Koh) | \| 1995-2005 \|  \| Hospital \| \| --- \| --- \| --- \| | South Korea  (Seoul) | Individualized | No | Yes | No | Yes | Serious |
| Masjedi (Tabarsi) | 2002-2006 | Iran | Standardized | No | Yes | No | Yes | Serious |
| Migliori (Centis) | 2001-2004 | Italy | Individualized | No | Yes | No | Yes | Serious |
| Mitnick (Mitnick) | 1996-2002 | Peru (Lima) | Individualized | No | Yes | No | Yes | Serious |
| Munsiff/Li (Ahuja) | 1992-1997 | USA (New York) | Individualized | No | Yes | No | Yes | Serious |
| Narita (Narita) | 1993-1997 | USA (Florida) | Individualized | No | Yes | No | Yes | Serious |
| O’Riordan (Pasvol) | 1982-2004 | UK (London) | Individualized | No | Yes | No | Yes | Serious |
| Palmero (Palmero) | 1996-1999 | Argentina (Buenos Aires) | Individualized | No | Yes | No | Yes | Serious |
| Perez-Guzman (Vargas) | 1994-1995 | Mexico | Individualized | No | Yes | No | Yes | Serious |
| Park (Seung) | 1998-2002 | South Korea  (Masan) | Standardized | No | Yes | No | Yes | Serious |
| Quy (Dang/ Cobelens) | 1998-2000 | Vietnam (Ho Chi Minh City) | Standardized (first line drugs only) | No | Yes | No | Yes | Serious |
| Schaaf (Schaaf) | 1998-2002 | South Africa | Individualized | No | Yes | No | Yes | Serious |
| Shin (Shin) | 2000-2004 | Russian Federation (Tomsk) | Individualized | No | Yes | No | Yes | Serious |
| Shiraishi (Shiraishi) | 2000-2007 | Japan (Tokyo) | Individualized | No | Yes | No | Yes | Serious |
| Tupasi (Quelapio) | 1999-2003 | Philippines | Individualized | No | Yes | No | Yes | Serious |
| Uffredi (Robert) | 1998-1999 | France (Paris) | Individualized | No | Yes | No | Yes | Serious |

*Included studies were all observational cohort studies. Treatment allocation was determined according to usual clinical practice in each setting. Bias in the selection of studies for inclusion in the individual patient data meta-analysis was unlikely, as patient outcomes in included studies was similar to that in studies that were not included. ** All studies were observational studies, therefore bias in the selection of patients for treatment (confounding by indication) cannot be excluded. Clinicians who assessed outcomes in the included studies were not blinded to treatment allocation. A detailed description of the outcome definitions for included in each study is found in a Supplement of Ahuja et al [Reference [19](#_ENREF_19) in the main manuscript].
